# Mode-Matched Resonant Excitation of a Nanowire Quantum Dot in a Nanophotonic Waveguide

**DOI:** 10.1021/acs.nanolett.5c04530

**Published:** 2025-11-24

**Authors:** Sayan Gangopadhyay, Lingxi Yu, Tarun Patel, Matteo Pennacchietti, David B. Northeast, Robin L. Williams, Philip J. Poole, Michael E. Reimer, Dan Dalacu

**Affiliations:** † Institute for Quantum Computing, 8430 University of Waterloo, Waterloo, Ontario N2L 3G1, Canada; ‡ Department of Physics and Astronomy, University of Waterloo, Waterloo, Ontario N2L 3G1, Canada; ¶ National Research Council of Canada, Ottawa, Ontario K1A 0R6, Canada; § Department of Physics, University of Ottawa, Ottawa, Ontario K1N 6N5, Canada; ∥ Department of Electrical and Computer Engineering, University of Waterloo, Waterloo, Ontario N2L 3G1, Canada

**Keywords:** nanowire quantum dots, single photons, resonance excitation, coherent control

## Abstract

Nanowire-based quantum dots as sources of single photons are promising candidates for the implementation of quantum photonic technologies. Achieving coherent control of these sources is essential for generating indistinguishable single photons—a key requirement for quantum interference. However, coherent excitation of nanowire quantum dots via resonant pumping has remained a long-standing challenge due to high laser suppression requirements. Here we establish a reliable technique to implement resonant excitation of a quantum dot in a tapered single-mode nanowire waveguide by complementing polarization–rejection with mode-matching to minimize the amount of backscattered laser. We demonstrate low multiphoton emission [*g*_*X*_^(2)^(0) = 0.019] and multiple Rabi oscillations under pulsed resonant excitation. We also report on two-photon indistinguishability under resonant excitation, achieving an interference visibility of 41%. This is a significant improvement over incoherent excitation and represents an important step in the development of a scalable approach for producing coherent single-photon sources.

## Graphical abstract

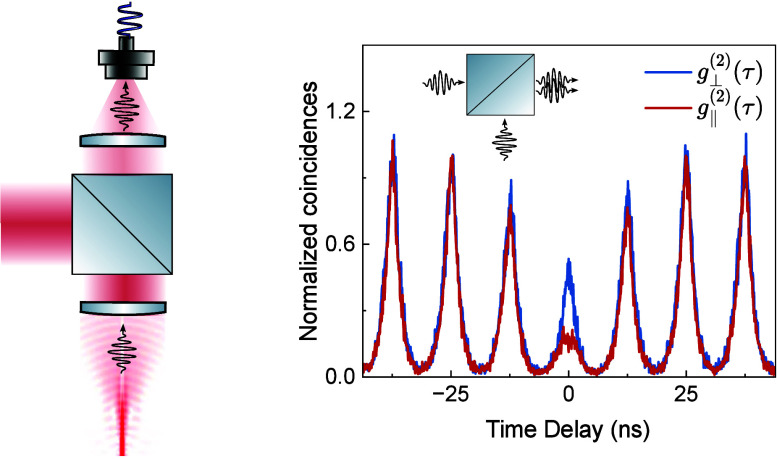

Quantum photonic technologies, including measurement-device-independent quantum key distribution,^1^ linear optical quantum computing^2^ and measurement-based quantum computing,^3^ require sources that generate single, identical photons on-demand with negligible probability of emitting multiple photons per trigger pulse.^4^ Resonantly excited epitaxial semiconductor quantum dots have been used to demonstrate the on-demand generation of such indistinguishable photons.^5–7^ As many sources will be required for the implementation of the above technologies, scalable architectures are desirable and InAsP nanowire quantum dots (NWQDs) incorporated within tapered InP photonic nanowires offer deterministic positioning^8^ with the added possibility of on-chip integration.^9,10^ Two-photon interference (TPI) measurements of NWQD sources, used to establish the distinguishability between emitted photons, have thus far been implemented using incoherent pumping.^11,12^ Exciting incoherently is known to introduce dephasing associated with charge noise^13^ and interactions with phonons,^14^ as well as excitation timing jitter associated with carrier capture processes.^15^ These act to limit observable TPI visibilities (*V*_TPI_) to values of *V*_TPI_ < 5% when the NWQD is excited above-gap at 50 ns intervals,^11^ increasing to *V*_TPI_ ∼ 9% for 12.5 ns pulse separation and to *V*_TPI_ ∼ 19% for excitation into the dot’s p shell.^12^ On the other hand, it is well established that resonant excitation leads to fewer interactions between a quantum dot with its environment and eliminates excitation timing jitter. Thus, to reveal the full potential of NWQDs as sources of indistinguishable photons, resonant excitation appears necessary.^5–7^

Resonant excitation based on collinear top-pumping schemes is typically implemented with cross-polarized excitation and collection arms.^16,17^ The pump laser, on reflection from a planar quantum dot sample, maintains its original linear polarization and is rejected by the analyzer in the detection arm. The residual orthogonal polarization has a “clover-leaf” pattern^18^ with highly suppressed intensity at the center, facilitating further rejection by coupling into a single-mode fiber which acts as a spatial filter. NWQDs, however, are embedded within nanophotonic waveguides with subwavelength lateral dimensions (∼250 nm) and large aspect ratios (>40).^19^ Achieving high rejection of the resonant laser in such structures is more demanding as it is not evident whether the pump laser will retain its polarization after being scattered, nor if the resulting spatial profile can be filtered by the core of a single-mode fiber. Previous attempts at resonantly exciting NWQDs were limited to simple resonance fluorescence (RF) linescans^20,21^ or our own work^22^ establishing single-photon emission and coherent control, both of which suffered from insufficient laser rejection. Measurements of the indistinguishability of photons generated by NWQDs under strictly resonant excitation have not been performed prior to this work.

In this work, we demonstrate resonant excitation and coherent control in an InAsP/InP NWQD by augmenting polarization rejection^16^ with mode-matching.^23^ By matching the numerical aperture (NA) of the focused input laser beam with that of the tapered nanophotonic waveguide in which the NWQD is embedded,^24^ the laser is optimally coupled into the fundamental mode of the waveguide. Under these mode-matched conditions the laser intensity required to reach full population inversion in the quantum dot is minimized, as is the amount of backscattered laser. The result is a decrease in the residual laser intensity to be rejected with the polarization and spatial filtering optics, manifesting in an enhanced RF-to-laser-background ratio. This allows for the observation of Rabi oscillations beyond 4π with a probability of multiphoton emission *g* ^(2)^(0) < 0.02 at full inversion. Under mode-matched conditions and resonant excitation pulses separated by 12.5 ns, we measure raw TPI visibilities of *V*_TPI_ = 41%, a significant improvement compared to nonresonant measurements.^11,12^ This value, however, still leaves the majority of the emitted photons distinguishable, suggesting that even with resonant excitation, there still exists significant dephasing in the NWQD system on the 12.5 ns time scale.

The NWQDs used in this study are ∼3-nm-thick sections of InAsP located at a height of ∼1.5 μm within 20-nm-diameter InP nanowire cores grown at lithographically defined positions on the substrate, see ref 25 for details. These cores are clad with an InP shell to define a nanophotonic waveguide^26^ that is adiabatically tapered from a base diameter *D* to a tip diameter of ∼20 nm with a taper angle of ∼1°. A scanning electron microscopy (SEM) micrograph of a typical device is shown in Figure 1a.

**Figure 1:**
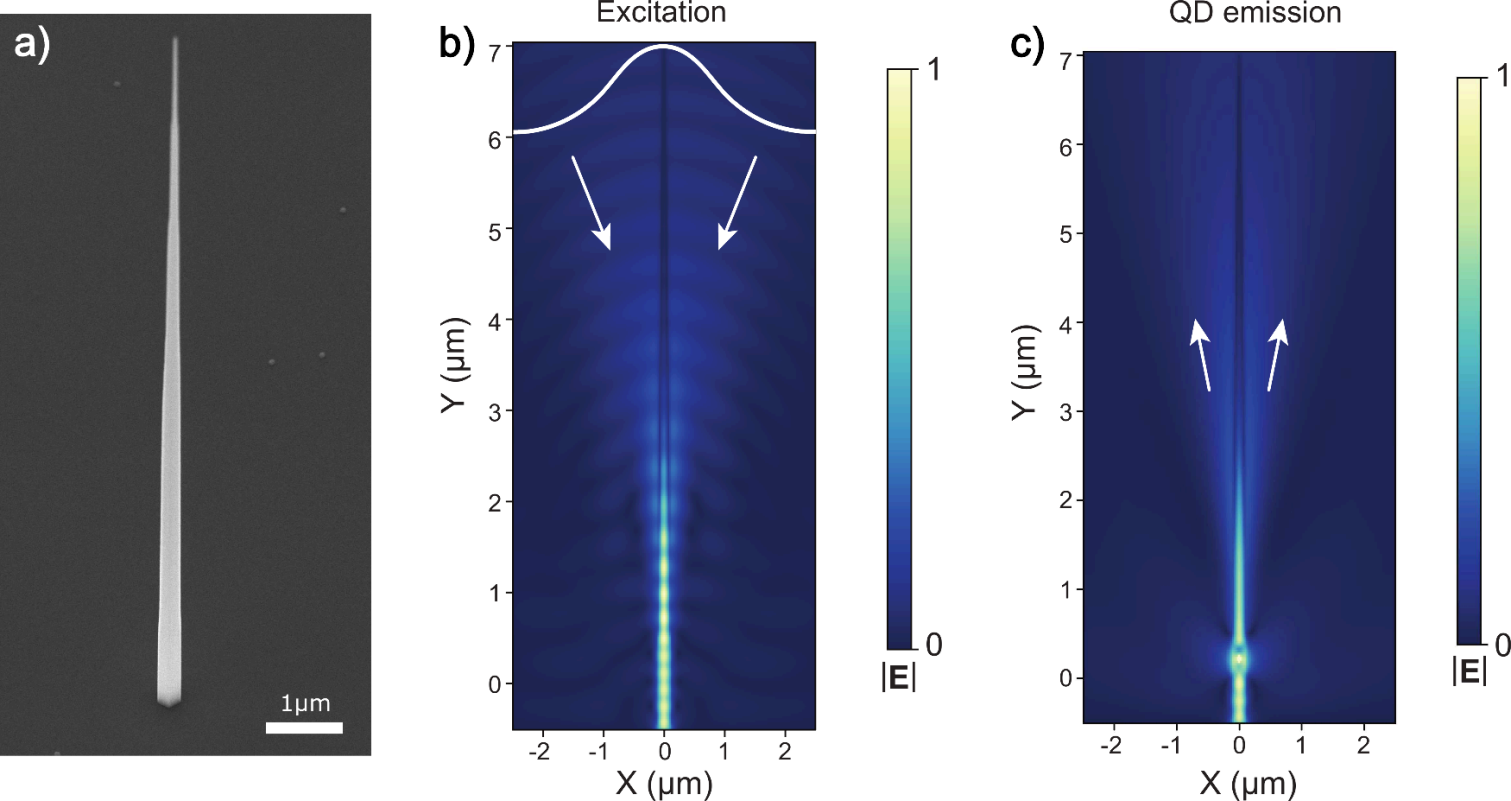
(a) SEM image of a single nanophotonic waveguide. FDTD calculations of the time-averaged electric field at a vertical plane of the waveguide during (b) in- and (c) out-coupling of the mode. In part b, the mode field diameter of the input beam was chosen to be 2.4 mm and focused using a lens with an NA of 0.81 and clear aperture of 4.75 mm. The focus of the input beam has been chosen to maximize the optical power coupled into the waveguide. The white arrows indicate that most of the light is out-coupled at a low NA of 0.4.

We target a nanophotonic waveguide diameter that supports only single-mode operation,^27,28^ i.e., *D* < λ/4 for an InP nanowire (*n* = 3.44), where λ is the emission wavelength. For waveguide diameters smaller than this single-mode cutoff, the emission of the quantum dot couples exclusively to the fundamental mode, after which the adiabatic taper expands the mode for coupling to a Gaussian far-field. We use finite-difference-time-domain (FDTD) calculations to simulate the in- and out-coupling for the nanowire waveguide (see the Supporting Information). In Figure 1c, we show FDTD simulations of the out-coupled dot emission from which we can extract an NA ∼ 0.4 for the taper geometry used here.^24^ For excitation, mode-matched coupling to the nanowire is achieved by the appropriate spatial tailoring of the incoming Gaussian beam (Figure 1b), and we expect >95% coupling into the fundamental HE_11_ mode. A detailed description of obtaining a perfect mode-match to the vertical nanowire waveguide from free space can be found in ref 29.

A photoluminescence (PL) spectrum of the s shell from the studied device (NWQD A) is shown in Figure 2a, where a pulsed 670 nm laser was used for excitation. The spectrum shows three distinct emission lines identified as the neutral exciton (X), the biexciton (XX), and the negatively charged exciton (X^–^), where peak identification is based on previous measurements of similar samples.^30^ The observation of two charged complexes in the time-integrated PL (X and X^–^) is a consequence of a quantum dot ground state that fluctuates between 0 and 1 electrons with the residual electron likely from unintentional background doping.

**Figure 2:**
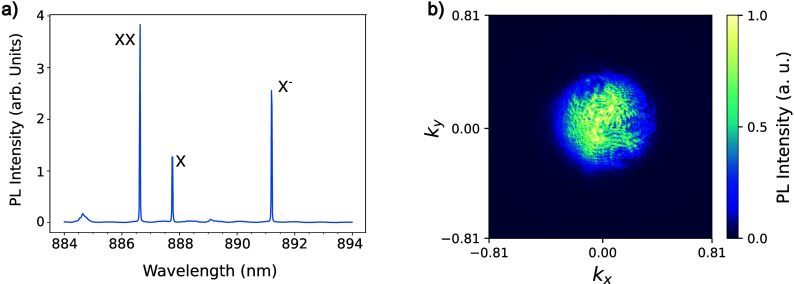
(a) PL spectrum of the s-shell emission of NWQD A. The three emission peaks are identified as the biexciton (XX), exciton (X), and negative trion (X^–^). (b) Far-field emission profile of the quantum dot s shell (all three emission lines) imaged using a 4-f Fourier microscopy setup. A Gaussian emission profile, with a divergence angle of ∼25° (NA = 0.42 ± 0.01), can be observed.

This s-shell emission is used to obtain information about the far-field emission pattern required to establish the mode-matching conditions. We use a 4-f Fourier microscopy setup (see the Supporting Information) to image the back-focal plane of the objective onto a CCD camera to form a Fourier, or far-field, image.^23^ To image just the s shell, a free space 20 nm band-pass filter centered at 890 nm is used to select only the X, XX, and X^–^ emission lines. The measured far-field of the s-shell emission of the investigated dot is shown in Figure 2b. We observe a Gaussian emission profile with a divergence angle of ∼25° (NA = 0.42 ± 0.01), consistent with single-mode operation of the nanophotonic waveguide.^24^ This NA value is used to tailor the mode field diameter of the pump beam such that the NA of the focused in-coupling laser beam matches that of the measured far-field emission.

For the resonant laser, we use a narrow wavelength slice from a 70 fs pulsed laser (resulting in ∼20 ps pulses), which is then coupled into a single-mode fiber. The laser is launched into free space using a fiber collimator and coupled into the excitation arm of an orthogonal-polarization-based dark-field confocal microscopy setup^16^ (see the Supporting Information). We choose a fiber collimator with focal length *f* = 11 mm to provide a mode field diameter of MFD ∼ 2.4 mm, which, for the cryogenic objective used in the experiments (NA = 0.81), gives an effective NA ∼ 0.42, matching the NA of the nanowire determined above. To verify the mode-matched coupling, we compare the far-field images measured with the polarization optics in the dark field microscope set to collinear, i.e., not rejecting the scattered laser. Figure 3a shows the far-field image where the laser is aligned to the nanowire but focused on the substrate, whereas in Figure 3b, the focus is set for mode-matching. Both images are measured with the resonant laser power set to 7 nW. From the two images, we calculate an extinction ratio $1-\frac{I_{\mathrm{mm}}}{I_{s}}=80\%$ , where *I*_mm_ (*I*_s_) are measured intensities for mode-matched (substrate) focus. Mode-matching, therefore, already provides a laser rejection of 5× even before applying the polarization rejection.

**Figure 3:**
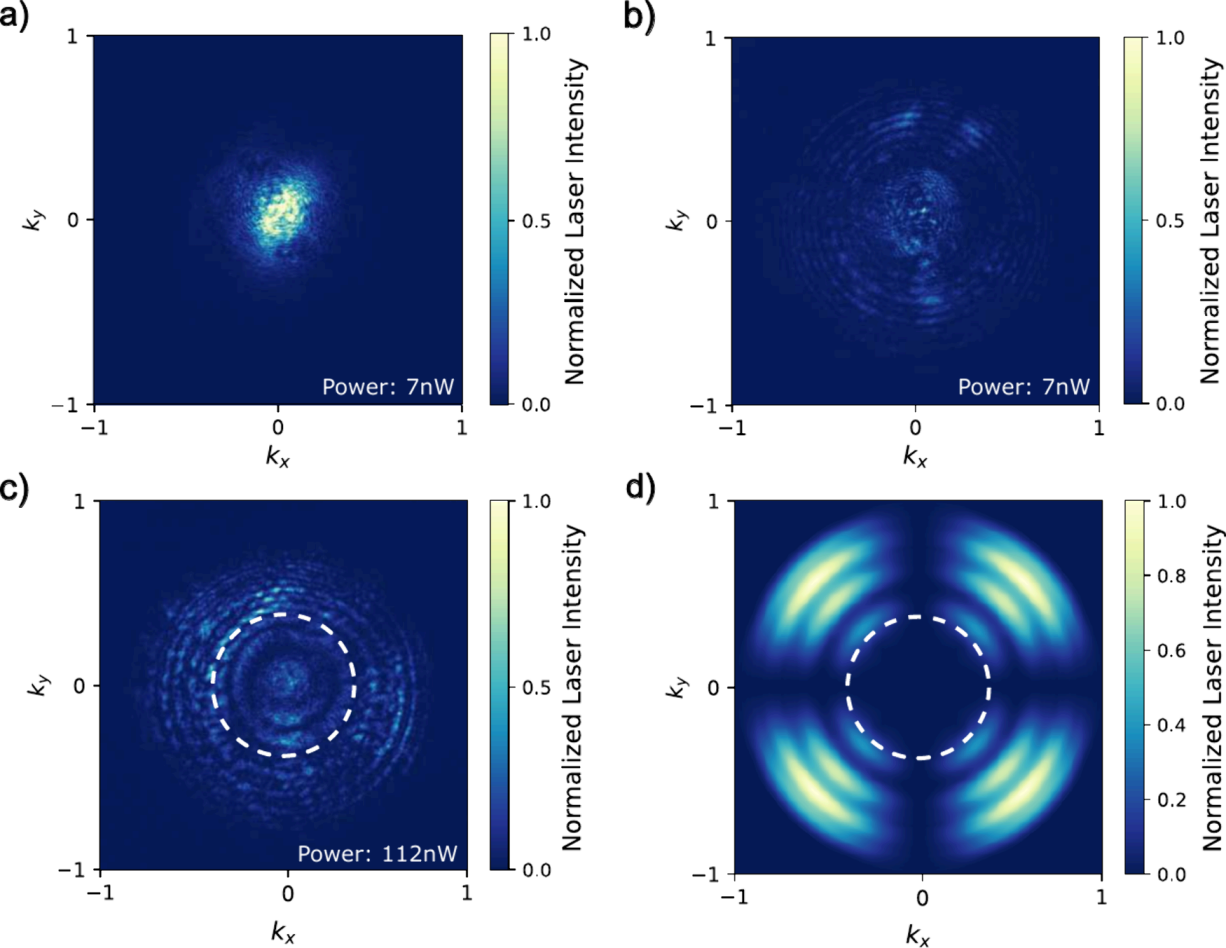
Far-field images of reflected laser intensity with 7 nW of laser aligned to the nanowire and (a) focused on the substrate or (b) focused for mode matching. In parts a and b, the polarization optics were set to collinear. (c) Same as part b but under cross-polarized conditions and at a laser power of 112 nW. (d) FDTD simulation of the expected angular distribution of the back-reflected laser under cross-polarized and ideal mode-matching conditions. The white dashed circles in parts c and d indicate the collection angle under mode-matching conditions.

For mode-matched RF alignment, we minimize the far-field intensity of the back-reflected laser under cross-polarized conditions. The measured far-field image is shown in Figure 3c, where the laser power has been increased by 17× to 112 nW for improved visibility on the camera. For comparison, Figure 3d shows a FDTD simulation of the expected angular distribution of the back-reflected laser with cross-polarization under ideal mode-matching conditions. The measured far-field reproduces some features seen in the simulation though not as well-defined and with more intensity at low NA, i.e., within the dashed white circle in Figure 3c, where the quantum dot emission is (Figure 2b). Absent is the clear reduction in intensity every 90° that defines the “clover-leaf” pattern. We speculate that the differences may be associated with nonuniform polarization scattering arising from steps and rotations of the crystal facets that define the taper as described by Versteegh et al. in ref 31 and which were not accounted for in the numerical calculations. We can also expect some blurring of the nanowire’s far-field pattern from parasitic scattering at the various optical surfaces in the setup since we are obliged to pump at high power just to register intensity on the camera.

Once mode-matched, the resonant laser is set to λ_X_ = 887.7 nm, corresponding to the neutral exciton, and the emission is coupled to a single-mode fiber to spatially filter out the larger *k*_*x*_ and *k*_*y*_ components. This is then sent to the fiber-coupled spectrometer. Exciting solely with the resonant laser, however, results in very little exciton emission and in order to increase the count rates, a weak above-band pump is required (see below). It was already clear from Figure 2a, where both X and X^–^ emission are observed in the time-integrated above-band PL, that there are two possible ground states for this dot: one in which the dot is empty and one where the dot contains a single residual electron. When excitation is solely resonant, it appears that the latter ground state becomes dominant, making the neutral X inaccessible to the resonant laser. Such pumping scheme-dependent ground states are common in solid-state two-level systems,^32–38^ a dependence that is associated with the presence of nearby traps and their pump-dependent population dynamics.^32^

Here we speculate that the addition of the weak above-band pump populates the dot with a single hole, which recombines with the residual electron (likely captured from a nearby defect or impurity level), leaving the dot empty, i.e., “photoneutralized”.^33^ With no residual carrier in the dot, the neutral X is accessible to the resonant laser and will remain so until another carrier is captured from the nearby trap. This capture rate will depend on the above-band excitation, and we use a 780 nm continuous-wave laser with a power that maximizes the RF intensity, while ensuring that at this power PL solely from the above-band pump is indistinguishable from the background intensity. We note that the supplemental above-band excitation, so-called “optical gating”, is both dot- and complex-dependent. In Supporting Information, we present a second quantum dot from the same growth chip (referred to as NWQD B), in which the X transition can be efficiently inverted using only the resonant laser. However, for observation of the X^–^ emission, the additional weak above-band excitation is required. This suggests that, unlike NWQD A, the natural ground state of NWQD B under resonant excitation is empty. It appears that, in the NWQD system, the specific distribution of carrier traps around each quantum dot plays an important role in determining the excitation-dependent ground state and the optical gating requirements during resonant pumping.

We show in Figure 4a the RF at a resonant laser power corresponding to a pulse area of π, where the above-band power has been optimized as described above. In contrast to the above-band spectrum shown in Figure 2a, only a single emission peak corresponding to the X photon is observed, as expected. To demonstrate coherent control, we plot in Figure 4b the integrated PL intensity of the X emission as a function of resonant laser power. We measure up to an excitation pulse area close to 5π and observe over two Rabi cycles. From a fit to the oscillations (see the Supporting Information), we extract an X population of |c_e_|^2^ = 0.849 ± 0.003 at under π-pulse excitation and a damping parameter ξ = 0.071 ± 0.002, which we assume is due primarily to exciton–phonon coupling.^39^

**Figure 4:**
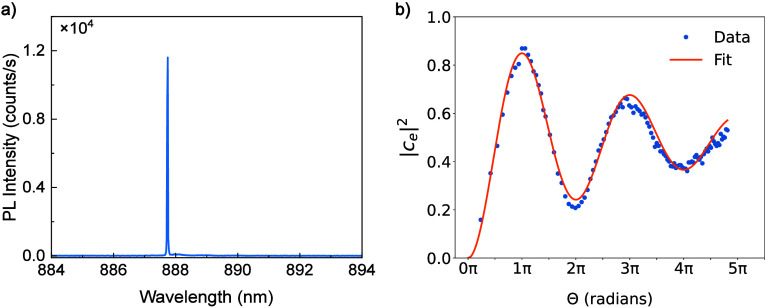
(a) RF spectrum of the neutral exciton, X, showing a single emission peak (compare Figure 2a). (b) Excited-state probability (|*c*_e_|^2^) as a function of the excitation pulse area. The model fit is described in the Supporting Information. At a π-pulse, we extract a maximum excitation probability of 0.84.

From the power-dependent RF measurements, we can quantify the efficacy of the laser rejection methodology. From the fit to the Rabi oscillations, we extract a laser power corresponding to a π-pulse of 3.5 ± 0.01 nW, approaching the calculated value of 2.07 nW under ideal mode-matching conditions (see the Supporting Information). This close to ideal implementation of mode-matching conditions provides the high laser rejection required to observe Rabi oscillations extending beyond 4π. By slightly detuning the laser away from resonance, we can estimate the signal-to-background ratio (SBR) under π-pulse excitation from which we obtain an SBR of 400:1.

We stress that the model fit shown in Figure 4b was performed on the raw data without any laser background subtraction. No corrections for laser leakage are required due to the high SBRs achievable under mode-matched conditions. To highlight the crucial role of mode-matching in these experiments, we have also measured the power-dependent RF in NWQD B for which mode-matching conditions could not be achieved. These measurements are shown in the Supporting Information. In this case, the fits to the Rabi oscillations required a background subtraction to account of resonant laser leakage.

The high laser rejection is verified by measuring the second-order correlation function, *g* ^(2)^(τ), using a Hanbury Brown & Twiss setup. The X emission is passed through an 850 nm long-pass filter and directed to two superconducting nanowire single-photon detectors (SNSPDs, 80% efficient, 100 ps jitter) via a fiber-coupled 50:50 beam splitter. Coincidences are recorded with a TimeTagger counting module. The left panel of Figure 5a shows the coincidence counts *g* ^(2)^(τ) obtained for both RF under π-pulse excitation as well as above-band excitation at 0.3 times the power required to saturate the transition, i.e., *P* = 0.3*P*_sat_. In both cases, the central peak is almost entirely absent (see the right panel) and we use the ratio of the counts in the central peak to the nearest neighbor peaks over a time window of 12.5 ns to quantify the single-photon emission. For above-band pumping, we obtain a value of *g* ^(2)^(0) = 0.0545 ± 0.001, and we attribute this small multiphoton probability to re-excitation of the dot from the same pulse.^40^ In the case of RF, coincidences in the zero-delay peak are reduced to *g* ^(2)^(0) = 0.0192 ± 0.001, demonstrating both high resonant laser rejection and reduced re-excitation events. We also observe a narrowing of correlation peaks under resonant excitation, due to a reduction in the excitation timing jitter. The elimination of timing jitter associated with exciton state preparation suggests that one can expect higher TPI visibilities, discussed below.

**Figure 5:**
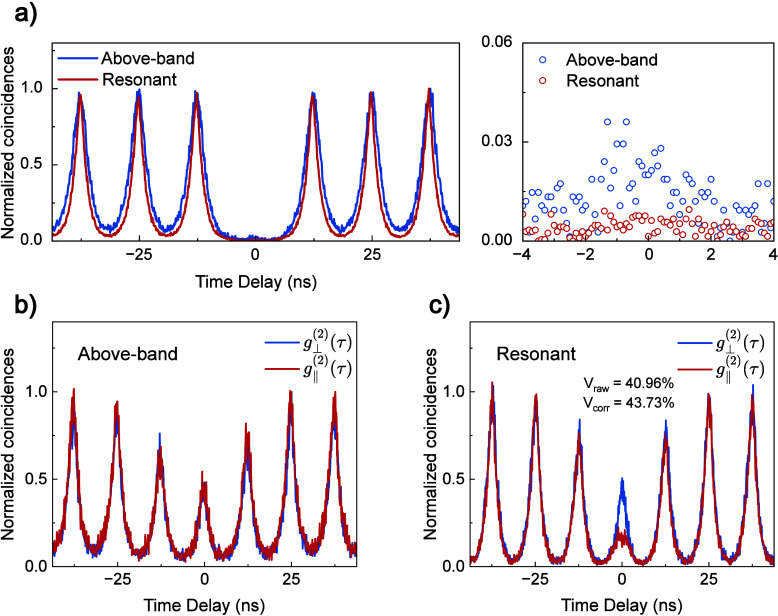
(a) Left panel: Second-order correlation, *g* ^(2)^(τ), of X under above-band (blue curve) and resonant (red curve) excitation. Right panel: Zoom of the coincidences registered in the zero-delay peak, demonstrating significantly lower multiphoton emission from RF compared to above-band excitation. Hong–Ou–Mandel interferometry measurements of X under (b) above-band and (c) resonant excitation. The red and blue lines represent correlation measurements under copolarized and cross-polarized configurations, respectively.

Finally, we verify the indistinguishability of the emitted photons with TPI measurements in a Hong–Ou–Mandel setup (see the Supporting Information). Sequentially emitted X photons separated by *T* = 12.5 ns, corresponding to the laser pulse separation, are filtered using a fiber-coupled 0.1 nm band-pass filter and interfered on the output beamsplitter of a *T* = 12.5 ns unbalanced Mach–Zehnder interferometer. The output ports of the beamsplitter are directed to the two SNSPDs, and coincidences are recorded for copolarized $g_{∥}^{\left(2\right)}\left(\tau \right)$ and cross-polarized $g_{⊥}^{\left(2\right)}\left(\tau \right)$ input photons. Parts b and c of Figure 5 show the co- and cross-polarized correlations for above-band and resonant excitation, respectively, where the characteristic variation in peak heights arises from the possible paths available to incident photons as described in ref 12.

The TPI visibilities are calculated using $V_{\mathrm{TPI}}\left(\tau \right)=\left[g_{⊥}^{\left(2\right)}\left(\tau \right)-g_{∥}^{\left(2\right)}\left(\tau \right)\right]/g_{⊥}^{\left(2\right)}\left(\tau \right)$ , and here we consider only the counts in the central peak: τ ± *T*/2 = 6.25 ns. Extracted visibilities using above-band excitation are negligible (*V*_TPI_ ∼ 0), increasing to *V*_TPI_ = 40.96 ± 1% under π-pulse excitation. If we correct for nonzero *g* ^(2)^(0) after ref 41, we obtain an intrinsic single-photon indistinguishability of $V_{\mathrm{corr}}=\frac{V_{\mathrm{TPI}}+g^{\left(2\right)}\left(0\right)}{1-g^{\left(2\right)}\left(0\right)}=43.73\%$ . This value represents a significant improvement compared with previous measurements on NWQDs where incoherent excitation was employed.^11,12^ It is still, however, substantially lower than state-of-the-art quantum dot sources operated under resonant excitation^17,42–44^ where near-unity visibilities have been demonstrated for 12.5 ns photon separation. These state-of-the-art sources are all Purcell-enhanced with radiative lifetimes less than a few hundred picoseconds, whereas here we are using an exciton with a much longer lifetime of *T*_1_ = 1.71 ± 0.02 ns (see the Supporting Information). We can therefore expect lower visibilities due to decoherence processes that act on this time scale, e.g., interactions with phonons, fluctuations of charge/spin environment, as has been previously reported, see ref 45.

From the expression^46,47^ *V*_TPI_ = *T*_2_/2*T*_1_, we calculate a coherence time of *T*_2_ = 1.398 ns using the measured values *V*_TPI_ = 41% and *T*_1_ = 1.71 ns. Using this *T*_2_, we obtain a pure dephasing time of *T*_2_^*^ = 2.36 ns from the relationship $\frac{1}{T_{2}}=\frac{1}{2T_{1}}+\frac{1}{T_{2}^{*}}$ . *T*_2_^*^ then accounts for the decoherence in the NWQD system (other than from the finite lifetime of the emitter, *T*_1_). One route to higher visibilities relies on increasing *T*_2_^*^ by, for example, employing electrical gating techniques to stabilize the charge environment^48^ or improving material quality through growth conditions.^49^

Alternatively, one can reduce *T*_1_ independently of *T*_2_^*^ by integrating the emitter with a high finesse optical microcavity.^50^ On the basis of the *T*_2_^*^ value observed here, we calculate a visibility of *V*_TPI_ > 90% for a Purcell-enhanced lifetime of *T*_1_ = 100 ps (see the Supporting Information).^51,52^ The fabrication of both gating and cavity structures around NWQDs is more difficult compared to other quantum dot systems due to the large aspect ratio of the nanowire devices and efforts to find nanowire-compatible approaches are ongoing.^53,54^ One promising avenue for integrating nanowires and cavities relies on a pick and place approach that we have developed for positioning nanowires on integrated photonic circuits.^12,55^ The approach works equally well with larger membrane-based photonic structures,^56^ and we envision picking up bullseye cavities,^57^ modified for compatibility with nanowire structures, and placing them directly on standing nanowires. Initial simulations (not shown) suggest Purcell enhancements of *F*_P_ > 25 from such hybrid devices with expected visibilities *V*_TPI_ > 95%.

To summarize, we have established a reliable technique to resonantly excite quantum dots embedded in adiabatically tapered single-mode nanophotonic waveguides. In particular, we have shown that mode-matching is crucial for effectively rejecting backscattered laser using a cross-polarization scheme. This has allowed us to observe high-visibility Rabi oscillations over 4π, important for future experiments involving coherent control of spins^58^ and the generation of linear cluster states^59^ from the quantum dot. It has also allowed us to benchmark the photon indistinguishability in the NWQD system under strictly resonant excitation (minimized excitation jitter and multiphoton emission), obtaining a TPI visibility of *V*_TPI_ = 41%. Together, these results mark an important milestone in the advancement of scalably fabricated sources of coherent photons.

## Supplementary Material



## Notes

Details on the experimental setup, FDTD calculations, modeling of Rabi oscillations, and measurements on additional NWQDs (PDF)
