# Supplementary material for: Mode-Matched Resonant Excitation of a Nanowire Quantum Dot in a Nanophotonic Waveguide
Source: Nano Lett. 2025 Nov 24;25(49):17096–103. doi: 10.1021/acs.nanolett.5c04530 (PMC12874642; doi:10.1021/acs.nanolett.5c04530)
Supplement: Supplementary file 1 [file nl5c04530_si_001.pdf]

Supporting Information: Mode-matched Resonant Excitation of a Nanowire Quantum Dot in a Nanophotonic Waveguide

Sayan Gangopadhyay[1,2,*,†,‡], Lingxi Yu[3,4,†], Matteo Pennacchietti[1,5], Tarun Patel[1,5], David B. Northeast[3], Robin L. Williams[2], Philip J. Poole[3], Michael E. Reimer[1,2,5], Dan Dalacu[3,4]

[1]Institute for Quantum Computing, University of Waterloo, Waterloo, Ontario, Canada, N2L 3G1.
[2]Department of Physics and Astronomy, University of Waterloo, Waterloo, Ontario, Canada, N2L 3G1.
[3]National Research Council of Canada, Ottawa, Ontario, Canada, K1A 0R6.
[4]Department of Physics, University of Ottawa, Ottawa, Ontario, Canada, K1N 6N5.
[5]Department of Electrical and Computer Engineering, University of Waterloo, Waterloo, Ontario, Canada, N2L 3G1.
[†]Equal contribution
[‡]E-mail: sgangopa@uwaterloo.ca
[*]Corresponding author

S.1 Optical setup

In the experiment, the nanowire quantum dot sample is mounted on a piezo-driven XY nano-positioner inside a standard closed-cycle cryostat and maintained at 4.2 K. Excitation beams are directed through a top window and focused onto the sample by a cryogenic apochromatic objective lens (Attocube LT-APO/NIR/0.81) mounted on a piezo-driven Z nano-positioner. The optical setup for resonant excitation (Figure S1a) is an adaptation of the polarization-based dark-field microscope designed by Kuhlmann et al. [1]. The laser light is sent to the microscope via a single mode PM fiber (780 HP, Thorlabs Inc) and collimated using an aspheric lens with a focal length $f = 11.0$ mm (60FC-4-A11-02, Schäfter + Kirchhoff GmbH). The linear polarization of the laser is ensured by passing through a polarizer (LPVIS050, Thorlabs Inc). The polarizer angle is set such that the s-polarized light is maximally reflected by a polarizing beamsplitter (PBS252, Thorlabs

Inc) towards the sample. The objective lens then focuses the laser onto the nanowire. The focal length of the collimating lens, $f = 11.0$ mm, is chosen to generate a collimated laser beam with a beam waist of 2.4 mm. This results in a partial filling of the aperture of the objective, creating an effective NA of ~ 0.42, closely matching the measured NA of the nanowire emission and thus ensuring mode matching of the input laser to the nanowire.

The p-polarized emission is transmitted through two PBSs which reject the s-polarized components of the back-reflected laser. A quarter-wave ($\lambda/4$) plate is used to optimize the polarization rejection by compensating for any birefringence introduced from the objective and from reflection off the sample. The p-polarized emission is coupled into a single mode PM fiber (780 HP, Thorlabs Inc) using an aspheric lens with focal length $f = 11.0$ mm (60FC-4-A11-02, Schäfter + Kirchhoff GmbH), ensuring mode-matching with the nanowire.

The optical setup used for $4-f$ imaging is shown in Figure S1(b). The objective lens creates a k-space or Fourier image of the sample at its back focal plane (BFP, located 12.5 mm behind the mechanical backside of the objective). An aspheric lens with $f =$ 20 mm is placed at a distance of roughly $2 \times f = 40$ mm away from the BFP. The 4-f system simply images the BFP of the objective on a TE-cooled CMOS camera (ZWO ASI585MC/MM Pro).

The optical setup used for HOM interferometry is presented in Figure S2. The quantum dot emission is launched into an unbalanced Mach-Zehnder interferometer (MZI) with a difference of 12.5 ns between the two arms. A PBS and half-wave ($\lambda/2$) plate are used to balance the intensity of light directed into either arm of the MZI through the reflection and the transmission ports of the PBS. The light propagating through the transmission port is immediately coupled into fiber and comprises the short arm of the MZI. The light propagating out of the reflection port of the PBS strikes a retro-reflector

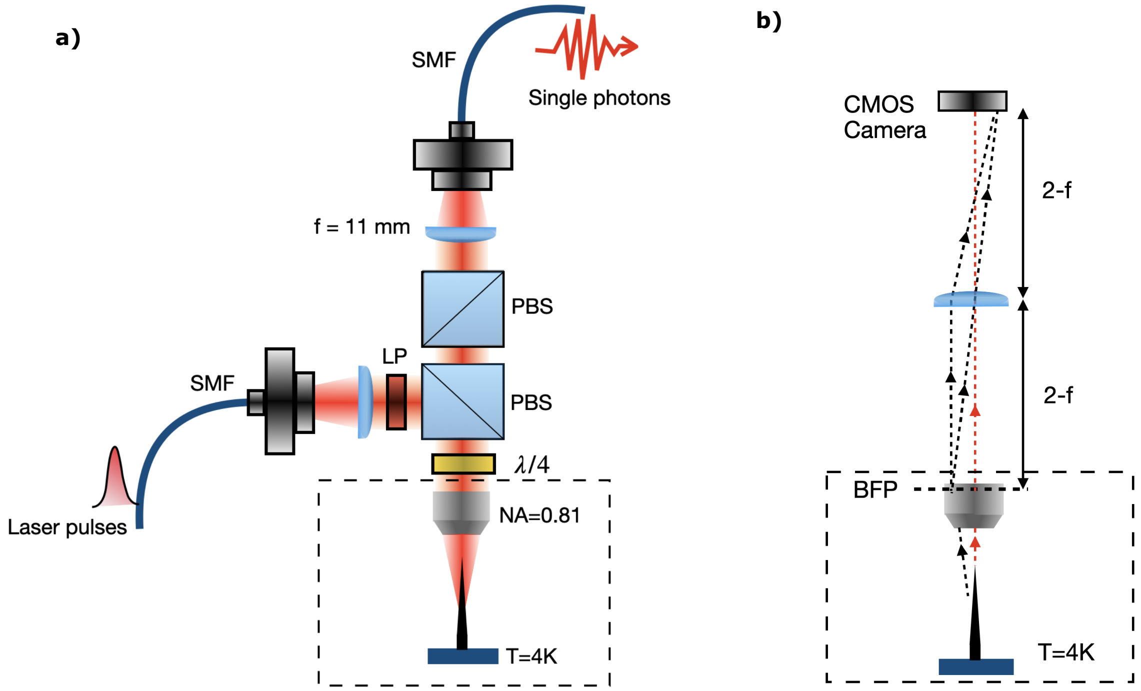

Figure S1: Schematics of (a) the dark-field microscope and (b) the $4-f$ system used in the experiments. Details are provided in the text.

mounted on a translatable stage. A fixed mirror is used to back-propagate the light to the PBS where it is now transmitted due to a double pass through the $\lambda/4$ plate. The longer free-space propagation compared to the transmission path of the PBS imparts a time delay of roughly 2.5 ns. The time delay can be adjusted by translating the retro-reflector back and forth. After fiber coupling, the light signal in the longer path receives an additional time delay of 10 ns in fiber with respect to the shorter path before arriving at a pigtailed beam splitter cube (50:50 BS). The output ports of the BS are sent to the two SNSPDs for detecting coincidences. The relative polarization of the two arms of the interferometer is controlled using the $\lambda/2$ plates at the inputs of the fiber couplers.

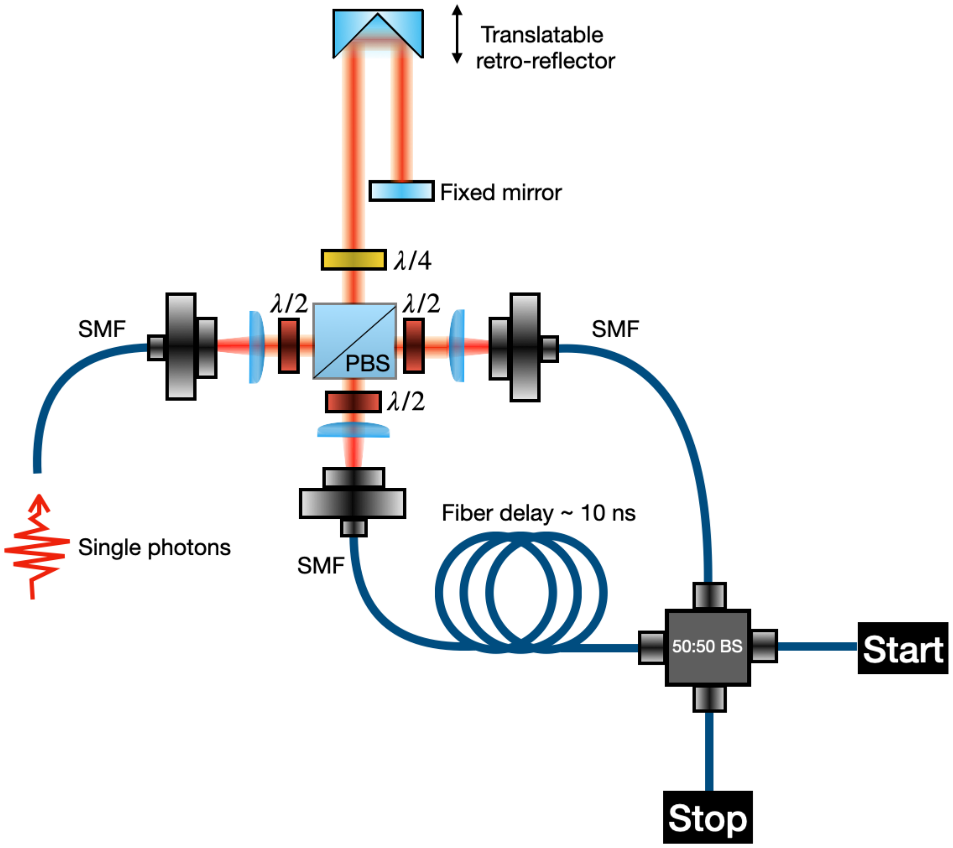

Figure S2: Schematic of the Hong-Ou-Mandel setup used for the two-photon interference measurements. Details are provided in the text.

S.2 FDTD calculations

The angular intensity distribution of the back-reflected laser was simulated using FDTD: 3D Electromagnetic Simulator [2] - a commercially available Maxwell's equation solver. The geometry of the nanowire used in the simulation was obtained from its scanning electron micrograph. The nanowire object comprises a cylindrical base and a conical taper with a top angle of 1.5°. A dielectric with an index of refraction $n = 3.44$ was used to simulate both the nanowire material (Wurtzite InP) and the Zinc Blende InP substrate. A Gaussian beam source [3] with a thin lens approximation was used to simulate the focused laser light. The thin lens was set to have an NA of 0.81, aperture diameter of

4.75 mm and an input beam waist of 2.4 mm. These parameters were used to accurately model the experimental setup. A power monitor was placed at the cylindrical cross section near the base of the nanowire. The focal spot of the Gaussian beam source was varied along the height of the nanowire and the optical power propagating through the nanowire was monitored. The location of the focal spot corresponding to maximum transmission through the power monitor was chosen for optimal mode-matching. For calculating the angular dependence of the back-scattered laser under mode-matched conditions, a box monitor [4] was used. The box enclosed both the nanowire and the Gaussian beam source but was placed above the substrate. The bottom facet of the box was disabled such that all the 5 remaining facets are in the same medium, i.e., air. This ensures that an accurate far-field projection can be computed. The angular dependence of the far-field projection using the box monitor has been plotted in Figure 3d of the main text.

S.3 Rabi calculations

We calculate the input resonant laser power corresponding to a $\pi-$pulse i.e. full inversion of the X transition [5]. We first estimate the dipole moment of this transition from the measured spontaneous emission rate. In the single mode operation of the nanowire, we assume that most of the quantum dot emission is funneled into the HE_{11} mode of the nanowire with the spontaneous emission rate given by[6]

$$\Gamma_{\mathrm{HE}_{11}} = 0.9 \times n\gamma_0 \tag{S1}$$

where n is the refractive index of the nanowire and γ_0 is the spontaneous emission rate in free space. We experimentally determine $\Gamma_{\mathrm{HE}_{11}}$ from time-resolved photoluminescence measurements and calculate $n\gamma_0$ using Eq. S1.

To determine the electric field required at the location of the quantum dot such that the area under an optical pulse is π, we start with the Wigner-Weisskopf theory of spontaneous

emission to calculate the transition dipole moment (μ) using the expression

$$n\gamma_0 = \frac{\mu^2\omega^3}{3\pi\epsilon_0\hbar c^3}. \tag{S2}$$

Substituting $n\gamma_0$ in Eq. S2 and rearranging, we find μ. We then use Rabi theory to obtain the electric field, $E(t)$, at the quantum dot for a given pulse area Θ:

$$\Theta = \int_{-\infty}^{\infty} \frac{\mu E(t')}{\hbar} dt'. \tag{S3}$$

We assume a Gaussian temporal pulse shape of the electric field, i.e. $E(t) = E_{\text{peak}} e^{-t^2/\tau^2}$, where τ is related to the full-width at half max by $\tau = \sqrt{2\ln(2)}\tau_{\text{FWHM}}$. Substituting $E(t)$ in Eq. S3 we get

$$\Theta = \frac{\sqrt{\pi}\mu E_{\text{peak}}\tau}{\hbar}, \tag{S4}$$

which we can rearrange to obtain E_{peak} at a $\pi-$pulse:

$$E_{\text{peak}} = \frac{\sqrt{\pi}\hbar}{\mu\tau} \tag{S5}$$

From FDTD calculations under mode-matched conditions (Section S.2), we determine the input laser electric field amplitude, E_{laser}, such that the field amplitude at the location of the quantum dot inside the nanowire is E_{peak}. In an empty simulation, i.e., without any material in the simulation region, we use the in-built `sourcepower(f)` function to calculate the total power injected into the simulation by the Gaussian beam source corresponding to a field almplitude of E_{laser}.

The optical power in a single pulse is given by

$$P_{\text{pulse}} = \texttt{sourcepower(f)}\,\tau\sqrt{\frac{\pi}{2}} \tag{S6}$$

The laser power resulting in a pulse power of P_{pulse} is then obtained by multiplying it by the repetition rate:

$$P_{\text{laser}} = P_{\text{pulse}} \times (80 \text{ MHz}) \tag{S7}$$

from which we obtain $P_{\text{laser}} = 2.07\,\text{nW}$ cited in the main text.

S.4 Rabi fitting

We use Ref. [5] to calculate the excited state population as a function of excitation laser pulse area. For continuous-wave resonant excitation, the time-dependent excited state population, $|c_e|^2$, is given by:

$$|c_e|^2 = \frac{1}{2(1+2\xi^2)}\left(1 - \left(\cos(\Omega' t) + \frac{3\xi}{\sqrt{4-\xi^2}}\sin(\Omega' t)\right)e^{-3\gamma t/2}\right) \tag{S8}$$

where $\Omega' = \Omega\sqrt{1-\xi^2/4}$, Ω is the Rabi frequency, $\xi = \gamma/\Omega$ and γ represents a damping parameter. Under pulsed resonant excitation, Ωt becomes the pulse area given by Eq. S4. By substituting $\Omega' t$ with $\Theta\sqrt{1-\xi^2/4}$ and $\gamma t = \Omega\xi t \to \Theta\xi$ into Eq. S8 we get the excited state population as a function of pulse area, Θ:

$$|c_e|^2 = \frac{1}{2(1+2\xi^2)}\left(1 - \left(\cos(\Theta) + \frac{3\xi}{\sqrt{4-\xi^2}}\sin(\Theta)\right)e^{-3\Theta\xi/2}\right). \tag{S9}$$

The scaling of Θ with the input power (P) is parameterized by k as $\Theta = k\sqrt{P-P_0}$ where P_0 is a constant background power. We numerically fit the recorded counts vs laser power data to an augmented model $C_{\texttt{exp}} = A \times |c_e(k, P_0, \xi)|^2$. The scaling factor A relates the normalized quantity $|c_e|^2$ to measured photon counts.

S.5 Resonant excitation of a second nanowire quantum dot

Resonant excitation on a second nanowire quantum dot, labeled NWQD B, was also performed. In this case, experiments were made on both the X and X^- transitions. Unlike NWQD A in the main text, here, no optical gating was required for observing X emission under resonant excitation. For the X^- transition, however, the additional weak above-band pump was necessary. This suggests that, contrary to NWQD A, this dot had an empty ground state under resonant excitation.

We note that for this nanowire, mode-matched conditions could not be achieved due to an insufficiently long taper which resulted in a non-Gaussian emission mode. Without

mode-matching, the resonant laser rejection was reduced such that the observed coherent driving was polluted by resonant laser leakage. To model the Rabi oscillations in the presence of residual resonant laser, we include a background intensity that scales linearly with laser power as a free parameter in the numerical fitting routine. The Rabi oscillations for both the X and X^- transitions in NWQD B are shown in Figure S3. We extract the probabilities of resonantly inverting the population of $|c_e|^2 = 0.920 \pm 0.004$ and $|c_e|^2 = 0.810 \pm 0.008$ for the X and the X^-. respectively.

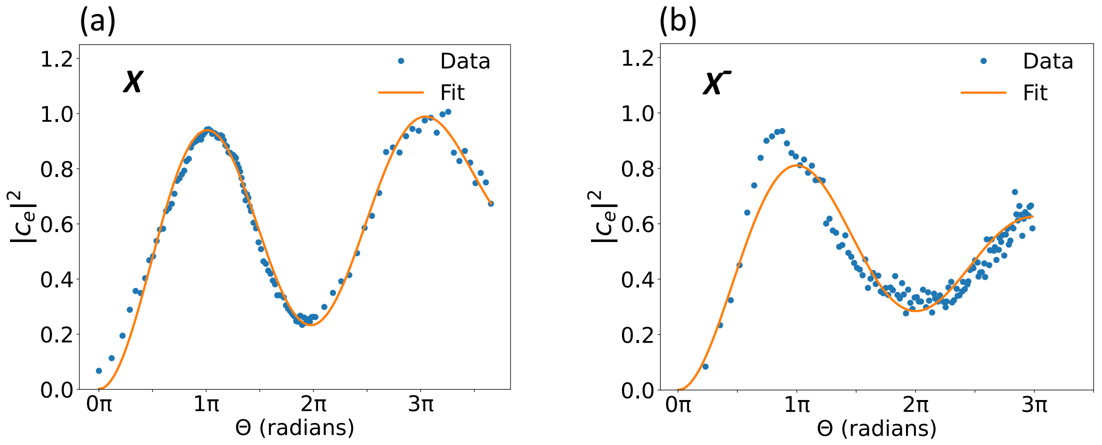

Figure S3: Excited state probability, $|c_e|^2$, as a function of excitation pulse area for (a) the neutral, X, and (b) charged, X^-, exciton transitions in NWQD B. Solid orange curves model fits after Eq. S9 with corrections for laser leakage.

Two-photon interference measurements were performed on the X and X^- transitions in NWQD B for both above-band and resonant excitation. Co- and cross-polarized correlations for the two transitions using the two excitation schemes are shown in Figure S4. For the X transition, similar visibilities as in NWQD A were observed, with V_{TPI} increasing from 0.8% for above-band excitation to 21.7% for resonant. For X^-, the visibility under above-band excitation was substantially higher, $V_{TPI} = 8.37\%$, but only increased to 14.35% when excited resonantly.

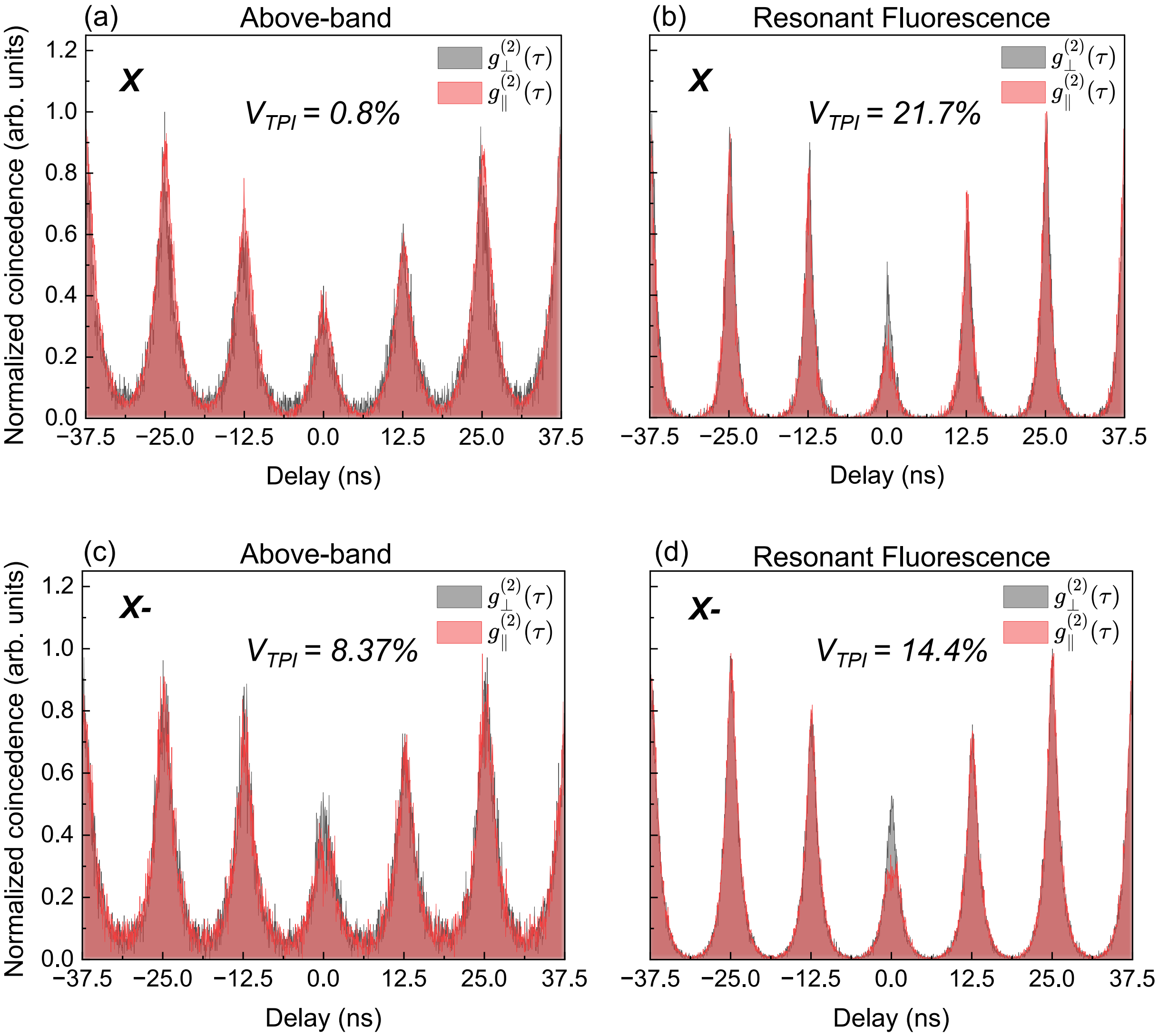

Figure S4: TPI measurements of (a,b) the neutral, X, and (c,d) the charged, X^-, excitons from NWQD B. In (a,c), the NWQD was excited above-band and in (b,d), excitation was resonant.

S.6 Additional measurements on NWQD A and NWQD B

Linewidths of the studied transitions were measured by scanning through the peaks using a piezo-driven fibre-based etalon with a 142 MHz bandwidth and 40 GHz free-spectral range. These high-resolution (HR) PL scans take tens of seconds, providing information on the coherence properties of the emitters on much longer time-scales compared to the

TPI measurements. The HRPL spectra from the three transitions are shown in Figure S5, taken at or close to P_{sat} ($\pi-$pulse) for above-band (resonant) excitation. In each case, only a single peak is observed: fine-structure splittings of the neutral excitons are assumed to be smaller than the linewidths and are not resolved. The linewidths, extracted from a Lorentzian fit for lineshapes with a negligible Gaussian component or else a Voigt fit, are listed in Table SI.

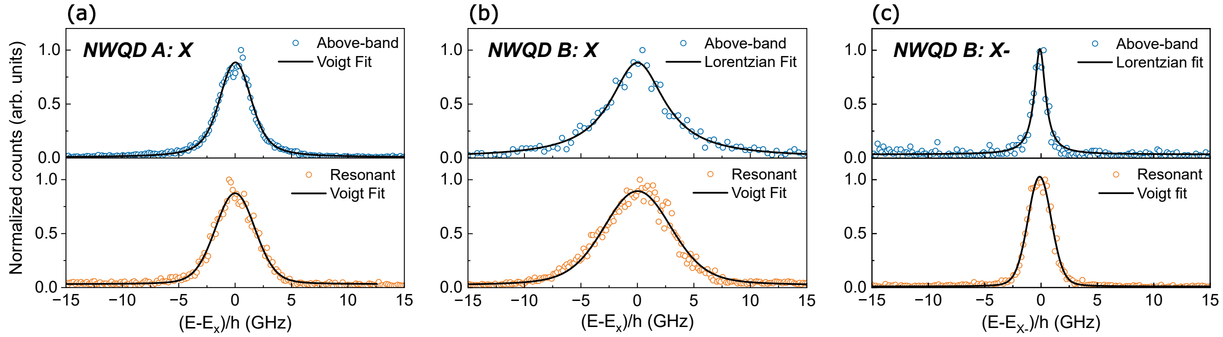

Figure S5: High resolution photoluminescence spectroscopy measurements on transitions in (a) NWQD A and (b,c) NWQD B using a scanning Fabry-Pérot etalon. Upper panels are measured using above-band excitation and lower panels using resonant excitation. Solid lines are Lorentzian or Voigt fits.

In all cases, a narrower linewidth is observed when exciting above-band, in particular for X^- in NWQD B where resonant excitation results in a $\sim 2\times$ increase and a switch from a Lorenztian to Voigt lineshape. Such behavior suggests that, at least on time-scales of a few tens of seconds, RF produces a noisier charge environment compared to above-band excitation. This is contrary to most studies of solid-state emitters where one typically observes broadening when illuminating non-resonantly, see, for example, Ref. [7]. Charge stabilization using non-resonant excitation is not, however, unprecedented, see Ref. [8].

We use the measured linewidths to estimate the coherence times, τ_c, from which we calculate the expected TPI visibilities using $V_{TPI} = \tau_c/2T_1$. These visibilities will inform on the achievable indistinguishability between sequentially emitted photons over the long

time-scales of the linewidth measurements. In general, assumed lineshapes are Voigt with linewidths, $\delta\omega_V$, given by[9]

$$\delta\omega_{\mathrm{V}} = 0.535\delta\omega_{\mathrm{L}} + \sqrt{0.217\delta\omega_{\mathrm{L}}^2 + \delta\omega_{\mathrm{G}}^2} \tag{S10}$$

where $\delta\omega_{\mathrm{L}} = \frac{1}{\pi T_2}$ is the Lorentzian contribution to the linewidth and $\delta\omega_{\mathrm{G}} = \frac{\sqrt{2\ln 2}}{\sqrt{\pi}T_{\mathrm{G}}}$ is the Gaussian contribution. Coherence times are then calculated using[10]

$$\tau_c = -\frac{T_G^2}{\pi^2 T_2} + \sqrt{\left(\frac{T_G^2}{\pi^2 T_2}\right)^2 + \frac{2T_G^2}{\pi}}. \tag{S11}$$

Excited state population decays, T_1, are extracted from the time-resolved PL traces, see Figure S6. The decays are measured using both above-band and resonant excitation, where the former includes a component associated with carrier relaxation processes[11]. For calculating the visibility, mono-exponential fits to the decays obtained using resonant excitation are used as they more closely approximate the radiative lifetime. Extracted lifetimes as well as TPI visibilities from both linewidth (long time-scale) and HOM (short time-scale) measurements are summarized in Table SI.

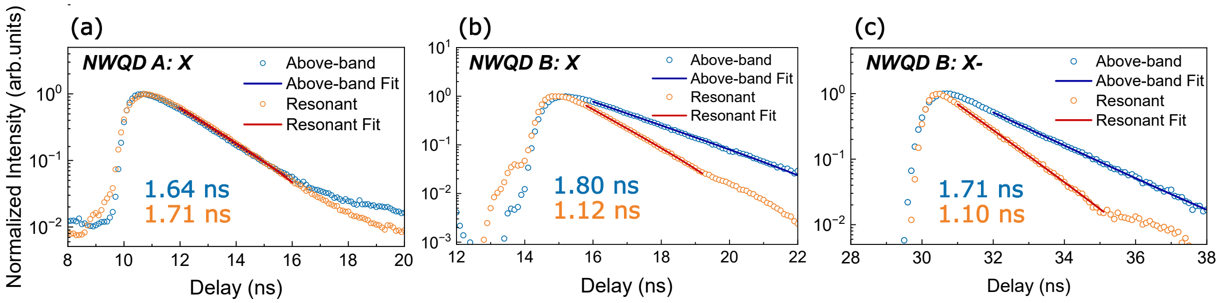

Figure S6: Time-resolved photoluminescence on transitions in (a) NWQD A and (b,c) NWQD B using above-band (blue symbols) and resonant (orange symbols) excitation. Solid lines are fits using a simple mono-exponential decay with extracted population decay constants shown in the figures.

The highest visibility observed was obtained using resonant excitation under mode-matched conditions. Visibilities are reduced when mode-matching is not achieved, pre-

	NWQD A: X		NWQD B: X		NWQD B: X^-	
	above-band	resonant	above-band	resonant	above-band	resonant
$\delta\omega$ (GHz)	3.38	4.19	5.87	7.16	1.18	2.46
T_1 (ns)	1.64	1.71	1.80	1.12	1.71	1.10
$\tau_c/2T_1$ (%)	5.7	3.9	4.7	3.6	2.5	1.0
V_{TPI} (%)	0	44	0.8	21.7	8.4	14.4

Table SI: Parameters extracted from the measurements. The linewidths shown in red (blue) text indicate Lorentzian (Voigt) lineshapes.

sumably due to increased spectral pollution from the resonant laser. Visibilities are also reduced when exciting non-resonantly, as expected. The less significant reduction in V_{TPI} observed for X^- in NWQD B (14.4% → 8.4%) is perhaps related to competition between a noisier charge environment using RF and significant relaxation jitter using above-band. Finally, there is a significant reduction in visibility when interfering over long time-scales (i.e. extracted from $\tau_c/2T_1$) but only when excitation is resonant.

S.7 Lifetime-dependent indistinguishability

In Figure S7 we calculate the expected improvement in indistinguishability with decreasing radiative lifetime of the emitter, T_1, using the relations $V_{TPI} = T_2/2T_1$ and $\frac{1}{T_2} = \frac{1}{2T_1} + \frac{1}{T_2^*}$, see Kim et al., Ref. [12]. For a pure dephasing time of $T_2^* = 2.36\,\text{ns}$, a visibility of $V > 90\%$ requires a reduction of the lifetime from $T_1 = 1.71\,\text{ns}$ (corresponding to the measured device, green star in the figure) to $T_1 = 100\,\text{ps}$. Such Purcell enhancements ($F_P \sim 17$) are readily achieved using bullseye cavities, see, for example, Ref. [13].

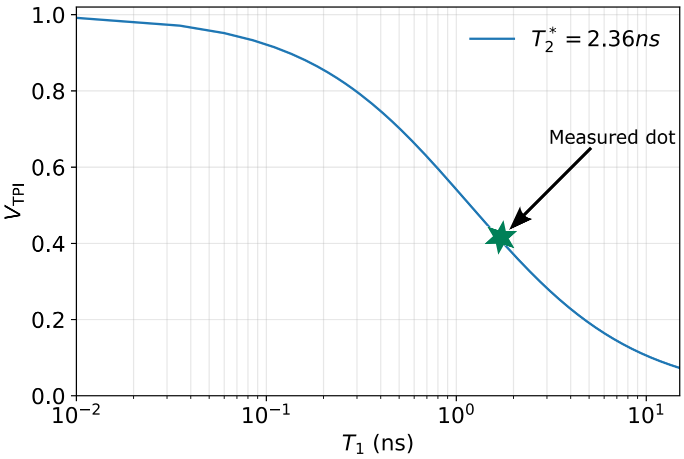

Figure S7: Dependence of the two-photon interference visibility on radiative lifetime of the exciton due to pure dephasing after Kim et al., Ref. [12].

References

[1] Kuhlmann, A. V. *et al.* A dark-field microscope for background-free detection of resonance fluorescence from single semiconductor quantum dots operating in a set-and-forget mode. *Rev. Sci. Instrum.* **84**, 073905 (2013).

[2] Lumerical inc.

[3] Plane wave and beam source simulation object. `https://optics.ansys.com/hc/en-us/articles/360034382854-Plane-wave-and-beam-source-Simulation-object`. Accessed: 2025-4-11.

[4] Far field projections from a box of monitors. `https://optics.ansys.com/hc/en-us/articles/360034915613-Far-field-projections-from-a-box-of-monitors`. Accessed: 2025-4-11.

[5] Fox, M. Resonant light–atom interactions. In *Quantum Optics*, 167–193 (Oxford University Press, 2006).

[6] Claudon, J., Gregersen, N., Lalanne, P. & Gérard, J.-M. Harnessing light with photonic nanowires: fundamentals and applications to quantum optics. *Chemphyschem* **14**, 2393–2402 (2013).

[7] Kuhlmann, A. V. *et al.* Charge noise and spin noise in a semiconductor quantum device. *Nat. Phys.* **9**, 570 (2013).

[8] Gazzano, O. *et al.* Bright solid-state sources of indistinguishable single photons. *Nat. Commun.* **4**, 1425 (2016).

[9] Yeung, E. *et al.* On-chip indistinguishable photons using III-V nanowire/SiN hybrid integration. *Phys. Rev. B.* **108**, 195417 (2023).

[10] Kambs, B. & Becher, C. Limitations on the indistinguishability of photons from remote solid state sources. *New Journal of Physics* **20**, 115003 (2018). URL https://dx.doi.org/10.1088/1367-2630/aaea99.

[11] Jahn, J.-P. *et al.* An artificial Rb atom in a semiconductor with lifetime-limited linewidth. *Phys. Rev. B* **92**, 245439 (2016).

[12] Kim, J. *et al.* Two-photon interference from an InAs quantum dot emitting in the telecom C-band. *Adv. Quant. Technol.* **8**, e2500069 (2028).

[13] Rickert, L. *et al.* High purcell enhancement in quantum-dot hybrid circular bragg grating cavities for GHz clock rate generation of indistinguishable photon. *ACS Photon.* **12**, 464 (2025).
